# Artificial Intelligence for Detection and Characterisation of Bone Metastases on MRI: A Scoping Review

**DOI:** 10.3390/cancers18172794

**Published:** 2026-08-28

**Authors:** Juncheng Huang, Wilson Ong Ying Fa, Aric Lee Wei Zheng, Timothy Shao Ern Tan, Gordan Toh Cheong Zheng, Ee Chin Teo, Jiong Hao Jonathan Tan, Naresh Kumar, James T. P. D. Hallinan

**Affiliations:** 1 Department of Diagnostic Imaging, National University Hospital, 5 Lower Kent Ridge Rd, Singapore 119074, Singapore; wilson.ong@mohh.com.sg (W.O.Y.F.); aric.lee@mohh.com.sg (A.L.W.Z.); gordantoh.cz@gmail.com (G.T.C.Z.); ee_chin_teo@nuhs.edu.sg (E.C.T.); 2 Department of Diagnostic and Interventional Imaging, KK Women’s and Children’s Hospital, 100 Bukit Timah Rd, Singapore 229899, Singapore; timothy.tan.s.e@singhealth.com.sg; 3 National University Spine Institute, Department of Orthopaedic Surgery, National University Health System, Singapore 119228, Singapore; jonathan_jh_tan@nuhs.edu.sg (J.H.J.T.); naresh_kumar@nuhs.edu.sg (N.K.); 4 Department of Diagnostic Radiology, Yong Loo Lin School of Medicine, National University of Singapore, 10 Medical Drive, Singapore 117597, Singapore; james_hallinan@nuhs.edu.sg

**Keywords:** artificial intelligence, machine learning, bone metastasis, magnetic resonance imaging

## Abstract

**Background/Objectives**: Bone metastasis is one of the most common manifestations of advanced malignancy and a major cause of morbidity, particularly when involving the spine. Magnetic resonance imaging (MRI) plays a central role in its detection and characterisation due to its high sensitivity for bone marrow infiltration. However, bone metastases may be missed on MRI, whilst interpretation can be time-consuming and challenging. The purpose of this study is to review and summarise the present evidence for artificial intelligence (AI) applications in the detection and classification of bone metastasis on MRI. **Methods**: A systematic, detailed search of the main electronic medical databases (PubMed, MEDLINE, Web of Science, and clinicaltrials.gov, last accessed on 1 January 2026) was undertaken in concordance with the PRISMA guidelines. **Results**: A total of 34 studies were included. AI applications were identified across several domains, including lesion detection, segmentation, disease classification, and predictive modelling. Deep learning approaches demonstrated strong performance for automated detection and segmentation, while radiomics-based models were frequently used for lesion differentiation and prediction tasks. Reported performance metrics were generally high, with area under the curve values commonly ranging from approximately 0.72–0.94, with most studies reporting AUCs exceeding 0.80 in internal validation, although substantial heterogeneity in study design, datasets, and validation strategies was observed. External validation and prospective evaluation were limited across most studies. **Conclusions**: Within the domain of bone metastasis, AI-based approaches have demonstrated encouraging performance and hold substantial potential to support clinical decision-making, including prognostication and prediction of treatment response. Nevertheless, further research is required to validate their clinical utility and to facilitate successful integration into routine clinical practice.

## Simple Summary

Bone metastases are a manifestation of advanced cancer. Magnetic resonance imaging (MRI) is highly sensitive for detecting bone marrow disease, but small or subtle metastases may still be overlooked. Image interpretation of multiple MRI images can be time-consuming. Artificial intelligence has emerged as a promising tool to assist radiologists in overcoming these challenges by improving lesion detection and classification. In this study, we systematically reviewed current research on artificial intelligence applications for identifying bone metastases on magnetic resonance imaging. Existing evidence suggests that these tools may enhance diagnostic accuracy and support clinical decision-making, including improved prediction of treatment response and complications. However, most studies remain limited in terms of generalisability, and further validation is needed before widespread clinical adoption. This work aims to clarify the current landscape and highlight future research priorities in MRI of bone metastasis.

## 1. Introduction

Bone metastasis is a major source of morbidity in oncologic patients, often presenting as part of advanced disease [1]. The skeleton is a frequent site of metastasis, thought to be due to its rich vascular network and supportive microenvironment for tumor cell colonization [2]. Some of the more common sites of primary malignancies which give rise to bone metastases include breast, prostate and lung cancers [3]. By the time osseous metastases become symptomatic, they may manifest as pain, pathological fractures or even spinal cord compression [4]. Their presence often signifies a poorer prognosis with more limited therapeutic options [5].

Imaging is important in the detection, characterization, staging, and follow-up of osseous metastases [6]. Conventional radiography remains widely available and cost-effective but is relatively insensitive, especially for early marrow-based disease [7]. On the other hand, computed tomography is regularly performed for staging and offers excellent cortical bone assessment, but it is less sensitive to early marrow infiltration [8]. While nuclear medicine techniques, such as bone scintigraphy and positron emission tomography (PET), allow for whole-body assessment and functional evaluation, they may lack specificity in the diagnosis of bone metastasis [9]. Beyond conventional imaging, artificial intelligence has also been explored in other aspects of bone oncology, including the characterization of bone tumours using spectroscopic techniques, highlighting the broader potential of AI in the assessment of skeletal malignancies [10]. In comparison to these methods, magnetic resonance imaging (MRI) has emerged as a key modality for evaluating osseous metastases due to its superior ability to detect bone marrow abnormalities [11]. It offers superior anatomical localization, serving as a particularly valuable imaging modality in assessing epidural extension and complications such as cord compression [12].

However, MRI assessment of bone metastases remains challenging, as image interpretation is time-consuming and requires careful evaluation across multiple sequences [13]. Lesion detection can be difficult, particularly in cases of subtle marrow infiltration or diffuse disease, and differentiation from benign mimics such as degenerative change, radiation therapy sequelae, infection, or red marrow reconversion adds further complexity [13,14]. Furthermore, manual delineation and quantification of disease burden are labor-intensive and subject to interobserver variability, hence limiting reproducibility [15].

Accurate classification and grading of osseous metastases are essential for treatment planning and prognostication, yet conventional imaging assessment remains challenging due to the heterogeneous appearance of lesions and variability across different primary tumour types [16,17]. Evaluating disease extent and accurately monitoring treatment response are also challenging, particularly in patients undergoing systemic therapies where imaging changes may be subtle or confounded by treatment-related effects [17]. In addition, prognostication requires integration of imaging with clinical and biological data [18].

In recent years, artificial intelligence (AI) has been increasingly explored as a potential solution to many of these challenges mentioned above [19]. Advances in machine learning and deep learning have enabled the development of algorithms capable of automated lesion detection and segmentation, diagnostic evaluation, severity grading, and even prognostication [20]. More recently, the emergence of large language models and multimodal AI systems has explored integrating imaging data with clinical information to support clinical decision-making [21]. However, despite these advances, clinical adoption remains limited by challenges related to generalisability, interpretability, and integration into existing workflows [22].

Hence, a comprehensive understanding of the strengths and limitations of AI in the MRI assessment of bone metastases is essential. In this review, we synthesise the current evidence, identify gaps in existing research, and highlight priorities for future research, with the aim of facilitating translation of AI technologies into meaningful clinical practice.

## 2. Materials and Methods

Given the anticipated large number of studies that would be extracted, a scoping review was performed to adequately represent the breadth and depth of the current literature.

### 2.1. Literature Search Strategy

We performed a literature search of the major databases (PubMed, MEDLINE, Web of Science, ClinicalTrials.gov) on 1 January 2026, according to the Preferred Reporting Items for Systematic Reviews and Meta-Analyses (PRISMA) guidelines. The PRISMA checklist can be found in Supplementary Table S1. The following medical subject headings (MeSH) and keywords were utilized: (“artificial intelligence” OR “AI” OR “deep learning” OR “machine learning” OR “convolutional neural network” OR “neural network” OR “radiomics”) AND (“bone” OR “osseous” OR “skeletal”) AND (“metastasis” OR “metastases”) AND (“magnetic” AND “resonance”). Limits were applied to include only English-language studies from the past 10 years, in order to capture the contemporary development of deep learning approaches in MRI while maintaining clinical relevance. The review protocol was registered in the Open Science Framework with ID 10.17605/OSF.IO/42FME.

### 2.2. Study Screening and Selection Criteria

A two-stage screening process was used. Studies were first screened independently by two authors (H.J. and W.O.) by title and abstract. A full-text review was then performed for any potentially eligible studies. Any discrepancies at either stage were reviewed by a third author (J.T.P.D.H.).

The inclusion criteria comprised studies evaluating the application of artificial intelligence to MRI assessment of bone metastases in human subjects, published in English. Although the search strategy primarily targeted spine-related MRI studies, studies evaluating skeletal metastases in other anatomical regions or using whole-body MRI were also included, provided the primary focus was on MRI-based AI applications for detection or characterisation of osseous metastases. Studies were excluded if they were non-original research (including review articles and editorial correspondence), unpublished work, conference abstracts, or case reports. Studies primarily focused on other imaging modalities (such as radiographs, CT, or nuclear medicine) were also excluded.

### 2.3. Data Extraction and Reporting

The selected studies were extracted and compiled into a spreadsheet using Microsoft Excel Version 16.82 (Microsoft Corporation, Washington, DC, USA). The following data were extracted:

1. Study details: authorship, year of publication and journal name;
2. Study aim;
3. Study details: Location of osseous metastasis, MRI sequences used;
4. Artificial intelligence technique used;
5. Key results.

## 3. Results

### 3.1. Search Results

**Figure 1:**
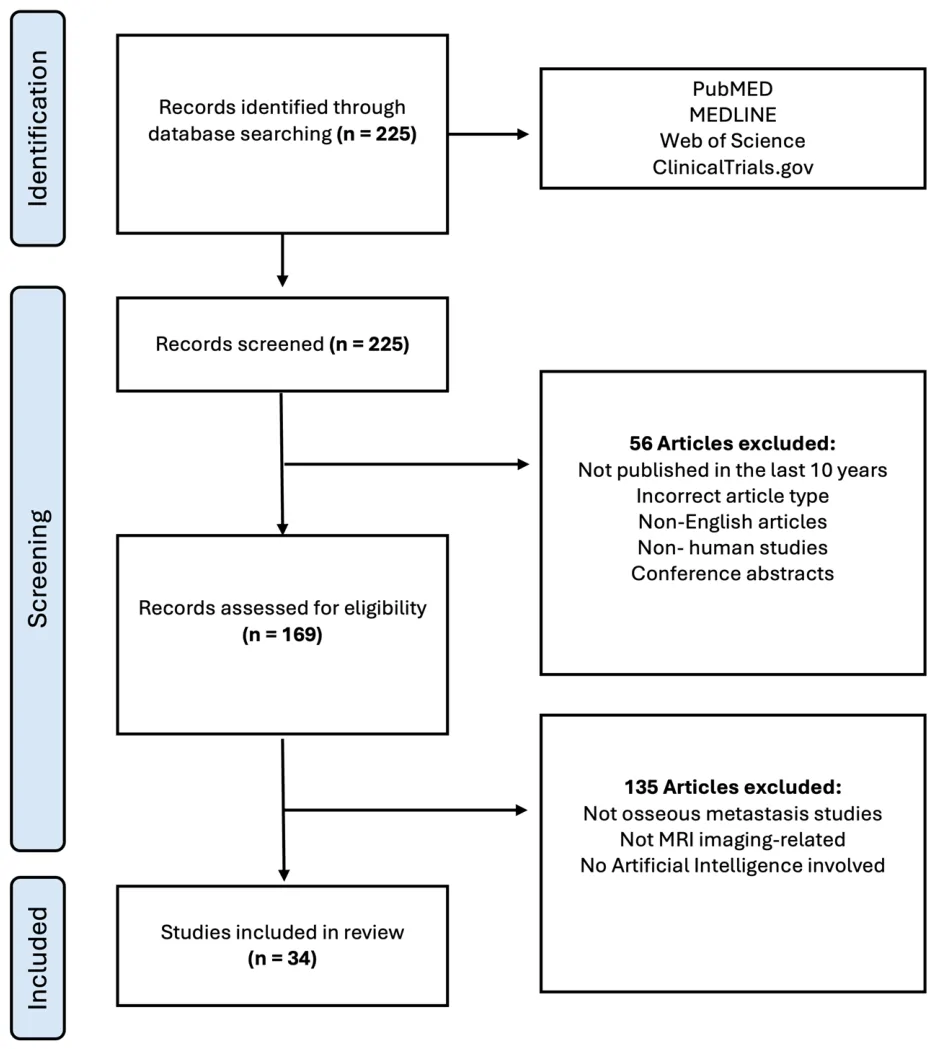
PRISMA flowchart showing the two-step study screening process. Adapted from PRISMA Group, 2020.

**Figure 2:**
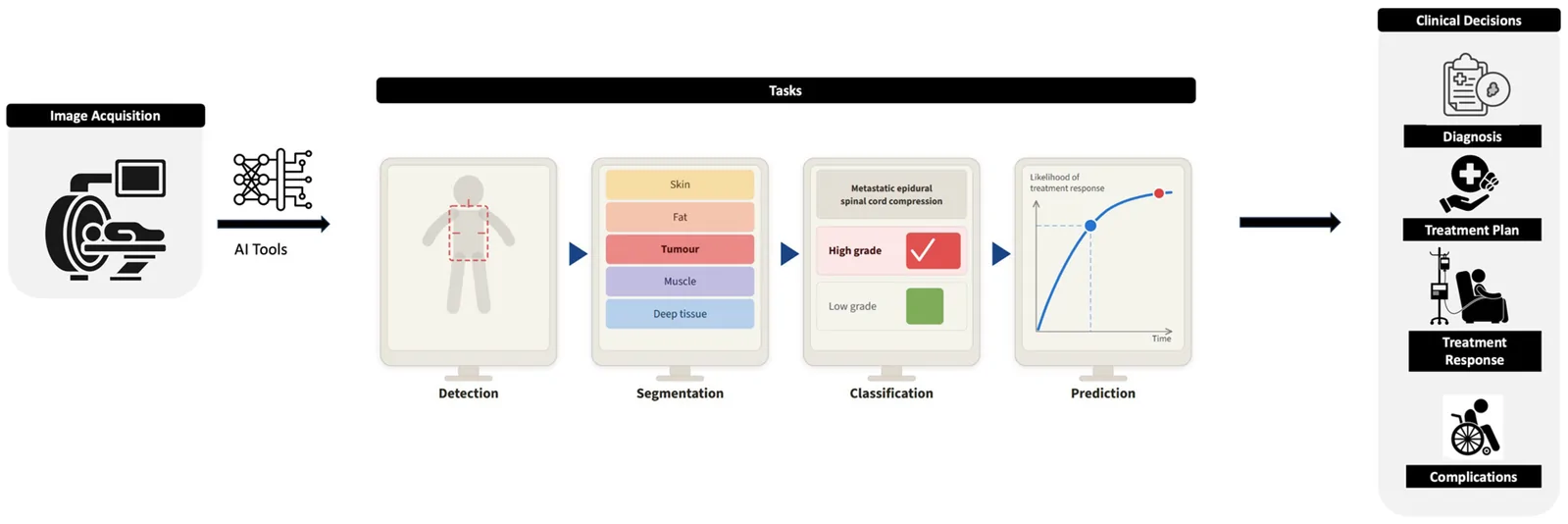
Overview of the MRI assessment pipeline for bone metastases and the corresponding applications of artificial intelligence (AI). AI methods support tasks including detection, segmentation, classification, and quantification, enabling improved diagnostic performance and clinical decision-making.

Our initial literature search identified 225 studies, which were screened according to the specified criteria. Following title and abstract screening, 56 studies were excluded due to ineligibility (including incorrect date range, inappropriate article type, non-English language studies, non-human studies, and conference abstracts). A further 135 studies were excluded after full-text review, as they were not relevant to osseous metastasis, MRI, or artificial intelligence. This led to 34 studies being included in the final analysis (see Figure 1 for the PRISMA flowchart), with their key characteristics summarized in Table 1. An overview of the MRI assessment pipeline for bone metastases and the corresponding applications of artificial intelligence has also been provided in Figure 2. Due to the heterogeneity of study designs, imaging protocols, and evaluation methods, a formal meta-analysis was not performed.

### 3.2. Segmentation

Segmentation represents a key application of artificial intelligence in the evaluation of bone metastases on MRI. It enables precise delineation of metastatic lesions, which facilitates objective quantification of disease burden [57]. Accurate segmentation is important for assessing tumour extent and improving reproducibility compared with manual measurements [58]. Recent studies have increasingly applied deep learning-based architectures, particularly U-Net models and variants, to automate segmentation tasks on MRI [59]. In particular, emerging developments such as self-configuring frameworks (e.g., nnU-Net) and weakly supervised learning approaches enable large-scale image analysis while unlocking large volumes of otherwise unusable data for training deep learning models [60]. This may reduce annotation burden and improve model generalisability, thereby supporting the development of new DL-based biomarkers.

Kim et al. (2024) developed a deep learning model for automated detection and segmentation of bone metastases on spine MRI using both 2D and 3D U-Net architectures [23]. The study utilised a large multicentre dataset comprising 662 MRI series from 302 patients, incorporating multiple MRI sequences including T1-weighted, contrast-enhanced T1-weighted, and fat-suppressed images. The model was trained and evaluated using different sequence combinations to optimise performance. In segmentation tasks, the 2D U-Net model using contrast-enhanced T1-weighted images demonstrated the best performance, with a Dice similarity coefficient of 0.699 and a pixel-wise recall of 0.653. The model also demonstrated promising detection capability, with per-lesion sensitivities of 0.828 and 0.857 in the internal and external test sets, respectively, outperforming the mean radiologist sensitivity (0.746) in the external dataset. These findings highlight the potential of deep learning models to provide reliable automated segmentation and concurrent lesion detection, supporting radiologists in identifying metastatic disease and improving workflow efficiency.

Despite these advances, several limitations remain. Reported segmentation performance is variable, with Dice scores generally in the moderate range, reflecting challenges such as lesion heterogeneity, small lesion size, and variability in MRI protocols. In addition, most models rely on labour-intensive manual annotations for training and are evaluated primarily in retrospective datasets, limiting generalisability [61]. These factors continue to pose barriers to clinical translation of automated segmentation tools.

### 3.3. Diagnostic Applications

Beyond detection and segmentation, artificial intelligence has increasingly been applied to improving diagnostic accuracy in the evaluation of bone metastases on MRI, particularly in distinguishing metastatic lesions from other pathologies with overlapping imaging features, such as multiple myeloma. These distinctions are clinically important, as they influence treatment strategies and prognostic assessment.

Several recent studies have utilized deep learning and advanced feature fusion strategies to improve diagnostic performance. Bian et al. (2026) developed a deep learning model [26] incorporating DenseNet169 feature extraction with Transformer-based feature fusion using conventional MRI sequences (T1WI, T2WI, and T2WI-fat suppressed (FS)). The study included a large multicenter cohort of 663 patients with osteolytic bone metastases or multiple myeloma. Among single-sequence models, T2WI-FS achieved the highest performance (AUC 0.765), while the Transformer-based fusion of T2WI and T2WI-FS sequences demonstrated the best overall results (AUC 0.783). Although statistical differences between models were not significant, the fusion approach showed improved clinical net benefit and good external generalizability.

Deep learning architectures have also been applied to capture complex imaging patterns associated with spinal metastases. Wang et al. (2023) designed a convolutional neural network model trained on MRI scans from 941 patients with spinal metastases [32]. Using a Softmax classifier, the model achieved an overall diagnostic accuracy of 96.45%, demonstrating strong potential for automated recognition of metastatic lesions and for supporting clinical evaluation of treatment response. While this high accuracy suggests strong potential for automated lesion recognition, such performance should be interpreted with caution given the lack of detailed external validation and potential dataset bias.

Radiomics-based machine learning approaches have also shown promising results in differentiating spinal metastases from other malignancies. Zhang et al. (2023) constructed an MRI-based radiomics nomogram incorporating both radiomic features and clinical factors from a cohort of 312 patients [35]. In the differentiation of spinal metastases from multiple myeloma, the radiomics model achieved AUC values of 0.853 in the validation set and 0.762 in the external test set, outperforming both a clinical-factor-only model and radiologist interpretation. Similarly, Liu et al. (2022) developed a vertebral MRI radiomics model to differentiate spinal metastasis from multiple myeloma, achieving an AUC of 0.84 using optimized radiomic feature selection [39]. Other deep learning frameworks include multi-view attention-guided networks, such as that proposed by Chen et al. (2022), which integrates sagittal, coronal, and axial MRI views for differentiation of spinal metastases (in the context of lung carcinoma) from multiple myeloma [40]. The model achieved an accuracy of 0.81 and an AUC of 0.78, outperforming both conventional radiomics approaches and radiologist assessments. These findings suggest that carefully selected radiomic descriptors can effectively capture subtle differences between metastatic lesions and multiple myeloma; however, reproducibility and robustness across centres remain ongoing challenges.

More recently, there has been a growing shift toward multimodal artificial intelligence models that integrate imaging data with complementary sources such as pathology, enabling more comprehensive disease characterization and improved diagnostic stratification. In particular, foundation models (FMs) leverage self-attention mechanisms and can process multimodal inputs, including images, text, audio, and video [62]. These models have the potential to streamline imaging workflows, enhance diagnostic accuracy, and support personalized treatment, while also using generative AI to produce synthetic images for augmenting limited real-world datasets. Ultimately, their effective deployment hinges on explainability, clinician trust, and smooth integration into existing clinical workflows.

Collectively, these studies demonstrate that AI-based classification models can enhance the differentiation of bone metastases from other skeletal pathologies on MRI. However, many of these studies are limited by retrospective study designs and relatively small or heterogeneous external validation cohorts, which may affect generalisability across institutions and imaging protocols. As newer models continue to demonstrate improved performance in such diagnostic tasks, they may play an increasingly important role in assisting radiologists, particularly in complex diagnostic scenarios.

### 3.4. Grading Disease Severity

Artificial intelligence has also been explored for grading the severity of complications associated with bone metastases, particularly in the assessment of epidural spinal cord compression (ESCC) [63], which is a critical factor in determining the urgency of surgical or oncologic intervention [64]. Accurate grading of ESCC on MRI is essential for treatment planning, yet conventional evaluation relies heavily on manual visual assessment by clinicians, which may be subjective and time-consuming. Furthermore, metastases to weight-bearing regions (such as the femur) can lead to an increased risk of pathological fractures [65].

Recent work has investigated automated methods to standardize and improve this process. Wang et al. (2025) developed an automated MRI-based system for grading spinal cord compression in patients with lung cancer spinal metastases [27]. The study utilized a dataset of 577 MRI slices with annotated multi-tissue segmentation masks and ESCC grades. Their approach employed a progressive pipeline combining segmentation and transfer learning-based classification, where models trained on related tasks are adapted to improve performance in a specific application. Initially, an encoder–decoder segmentation network, a deep learning architecture that extracts image features and reconstructs them into spatial maps, enhanced with a pyramid pooling module to capture information at multiple spatial scales and cross-attention mechanisms to prioritise relevant image regions, was used to segment key anatomical structures relevant to compression, including the vertebral body, spinal cord, and metastatic tumour tissue. Subsequently, a dual attention network (DAN), incorporating position and channel attention modules to weight spatial and feature importance within the image, was applied for classification. The segmentation model achieved an average pixel accuracy of 0.819 and an intersection-over-union score of 0.711, which reflects the degree of overlap between predicted and reference segmentations, indicating reliable identification of relevant anatomical structures. Using these extracted features, the classification system achieved an ESCC grading accuracy of 0.95 with a precision of 0.89, demonstrating strong performance in predicting clinically relevant compression grades.

Such findings suggest how AI-driven grading systems could improve the timely identification of patients at risk of significant neurological compromise, thereby facilitating appropriate surgical or medical interventions [66]. Moving forward, automated grading of disease severity is likely to play a crucial role in supporting clinical assessment of bone metastases. For example, AI systems can automatically identify lesions [67] and even trigger real-time alerts to the appropriate clinical teams [68] thereby enabling improved triage efficiency for emergencies such as pathological fractures or metastatic spinal cord compression.

### 3.5. Prediction

A significant proportion of studies in our review focused on predictive applications of artificial intelligence. To provide a clearer overview, these studies are categorised below according to the type of predictive task evaluated.

#### 3.5.1. Predicting Tumor Origin

Determining the primary origin of metastatic lesions is an important component of oncologic evaluation. Although many patients with spinal metastases have an established primary malignancy, the origin may occasionally remain uncertain, particularly when multiple potential primaries are present [69]. Conventional MRI characteristics can overlap across tumor types, thereby limiting diagnostic specificity [70]. Artificial intelligence, including deep learning and radiomics approaches, has therefore been explored as potential tools to identify subtle imaging patterns that may reflect the biological origin of metastatic lesions, including molecular subtyping (e.g., EGFR mutation status).

One such study by Duan et al. investigated whether radiomics and deep learning methods could identify the origin of spinal metastases using contrast-enhanced T1-weighted MRI [34]. In a retrospective study including 173 patients, two predictive models were developed: a radiomics-based model and a deep learning model. The results demonstrated that the deep learning model consistently outperformed the radiomics model across all datasets. In the internal training cohort, the deep learning model achieved an accuracy of 0.93 with an area under the receiver operating characteristic curve (AUC) of 0.94, compared with 0.84 and 0.93 for the radiomics model, respectively. In the internal validation cohort, the deep learning model achieved an accuracy of 0.74 and AUC of 0.76, while the radiomics model achieved an accuracy of 0.72 and AUC of 0.75. Similar trends were observed in the external test cohort, where the deep learning model achieved an accuracy of 0.72 and an AUC of 0.76, outperforming the radiomics model (accuracy 0.69, AUC 0.72). Notably, the deep learning model also surpassed expert radiologist assessment in the validation cohort, where human readers achieved an accuracy of 0.65 and an AUC of 0.68.

These findings suggest that deep learning models may capture complex imaging features associated with the biological origin of metastatic lesions, which may not be easily discernible through gross visual assessment. As such techniques continue to mature, AI-driven tools may serve as useful adjuncts in situations where the primary tumor origin is unclear, potentially guiding diagnostic evaluation and supporting more tailored oncologic management strategies.

#### 3.5.2. Predicting Treatment Response

Radiomics and artificial intelligence approaches have shown promising capability in predicting treatment response in patients with vertebral and skeletal metastases [71]. By extracting quantitative imaging features, these models could enable earlier and more objective evaluation of therapeutic outcomes compared with conventional radiologic assessment [72]. For example, Shi et al. evaluated radiomics in osteolytic spinal metastases from breast cancer and found that models based on ADC and fat-suppressed T2-weighted imaging (with regions of interest (ROIs) drawn manually by two radiologists) achieved strong predictive performance for chemotherapy response (AUC up to 0.905), outperforming conventional diffusion parameters [41]. Similarly, Candito A et al. developed an AI-driven software tool using whole-body diffusion-weighted MRI to automatically quantify metastatic disease burden in advanced prostate cancer. Their approach generated quantitative biomarkers such as total diffusion volume (TDV) and global ADC, which achieved an overall treatment response assessment accuracy of 80.5%, sensitivity of 84.3% and specificity of 85.7% [50].

Such findings suggest that AI-derived quantitative imaging biomarkers may provide clinically valuable insights into treatment response, and therapeutic decision-making in metastatic skeletal disease. By enabling earlier and more accurate prediction of response, these approaches may facilitate more timely and personalised management strategies for patients with bone metastases.

#### 3.5.3. Predicting Treatment Complications

Radiomics has also demonstrated potential in predicting treatment-related complications in patients with osseous metastases, enabling improved pre-procedural planning and risk stratification. By extracting quantitative imaging features from preoperative MRI, radiomics models may help identify patients at higher risk of adverse surgical outcomes that are otherwise difficult to anticipate using conventional clinical parameters alone [73].

For example, a study by Zhao WL et al. investigated the use of MRI-based radiomics models to predict hidden blood loss (HBL) in patients undergoing surgery for spinal metastases [30], which is a common but often under-recognised complication associated with poorer postoperative outcomes. In their retrospective cohort of 202 patients, radiomics features were extracted from sagittal T1-weighted and fat-suppressed T2-weighted MRI sequences acquired on both 1.5T and 3.0T scanners. Patients were stratified into high and low HBL groups using a 1000 mL threshold. Three types of models were compared: (1) the radiomics score (Radscore) model, chosen from the optimal radiomics model; (2) a clinical model; and (3) a combined model (combining the Clinical model and Radscore model). The Radscore model performed slightly better than the clinical model and the combined model, achieving an AUC of 0.809. Of note, among the different radiomics models, the fusion model (incorporating both T1-weighted and fat-suppressed T2-weighted features) demonstrated the highest predictive efficacy (AUC: 0.744).

These findings suggest that MRI-based radiomics may serve as a non-invasive tool for identifying patients at increased risk of perioperative complications. Early risk stratification may support more informed surgical planning and optimisation of perioperative management strategies. Future studies should also further explore the role of radiomics and AI in predicting fracture risk and treatment-related complications, such as cement leakage following vertebroplasty, which may further enhance procedural planning and patient selection.

#### 3.5.4. Artificial Intelligence in Whole-Body MRI for Bone Metastasis

Whole-body MRI (WB-MRI) has emerged as a powerful modality for systemic assessment of skeletal metastases, particularly in prostate cancer and haematologic malignancies. However, WB-MRI examinations generate large imaging datasets that may be time-consuming and challenging to interpret in routine clinical practice [74]. Artificial intelligence methods have been increasingly explored to assist in automated lesion detection and quantification across the entire skeleton [75], although there is overall limited evidence on the role of WB-MRI for evaluation of skeletal metastasis. This is possibly related to the challenges posed by limited availability, acceptance by patients, radiologists and health authorities, as well as longer acquisition times, although recent advancements have sought to reduce the scan times to less than 30 min [76].

Ceranka et al. developed a computer-aided detection system using multiparametric WB-MRI with a U-Net architecture, achieving automated segmentation of metastatic bone lesions with a Dice coefficient of 0.53 and detection sensitivity of 63% [37]. Similarly, Candito et al. proposed an AI-driven framework for automated quantification of tumour burden on diffusion-weighted WB-MRI, generating biomarkers such as total diffusion volume and global apparent diffusion coefficient for treatment response assessment in metastatic prostate cancer [50]. These approaches highlight the potential role of AI in facilitating quantitative whole-body disease assessment and longitudinal monitoring.

## 4. Discussion

Artificial intelligence applications in MRI have demonstrated considerable potential to enhance the detection and classification of bone metastases. Advances in computational techniques have enabled the extraction and analysis of complex imaging features that may not be readily appreciable through conventional radiological assessment. As demonstrated across the studies included in this review, AI-based approaches have been applied to a broad spectrum of tasks, including lesion detection, segmentation, disease grading, and clinically relevant prediction tasks. Collectively, these developments highlight the growing role of data-driven imaging analysis in supporting clinical decision-making and augmenting the diagnostic utility of MRI in metastatic bone disease.

The integration of AI into MRI interpretation may also help address several practical challenges encountered in routine radiology practice. Increasing imaging volumes and the complexity of oncologic imaging place substantial demands on radiologists [77], particularly in the evaluation of metastatic disease, where subtle lesions and diffuse skeletal involvement may be difficult to assess consistently. AI-assisted tools have the potential to streamline image interpretation through automated detection and longitudinal disease monitoring [78]. Such systems may improve efficiency and reproducibility [79], thereby allowing radiologists to devote greater attention to complex diagnostic reasoning and multidisciplinary patient care.

While these developments are promising, important considerations remain regarding the translation of AI technologies into clinical practice. The clinical value of AI tools must be carefully evaluated across the entire imaging pathway [80], from lesion detection and characterization to treatment planning and prognostication. In particular, the impact of AI on workflow integration, reporting practices, and clinical decision-making remains incompletely defined and requires further investigation [81]. A key challenge is to maintain model performance across disease presentations. In bone metastases, this includes distinguishing small-volume lesions from normal marrow, recognising diffuse marrow infiltration without discrete lesion boundaries, and differentiating residual or recurrent disease from treatment-related marrow changes, such as those following radiotherapy. In the multi-parametric whole-body MRI computer-aided diagnosis system developed by Ceranka et al., per-lesion sensitivity was only 63%, with a mean of 6.44 false positives per image and a lesion-level Dice coefficient of just 0.53 [37]. In particular, diffuse disease was excluded during annotation because metastatic involvement lacked well-defined boundaries, highlighting a limitation of lesion-based segmentation approaches in quantifying diffuse marrow disease. These challenges highlight the need for robust external validation across diverse clinical settings before such models can be considered sufficiently generalisable for routine clinical use.

A further limitation of the current literature is the focus on task-specific models, typically developed for a single application such as detection, segmentation, or classification. While effective, these approaches are difficult to scale and integrate into routine workflows. There is increasing interest in more generalisable approaches, including foundation and multitask models, which aim to handle multiple tasks within a single framework [82]. However, their application in musculoskeletal and oncologic MRI remains at an early stage.

Given the rapid evolution of AI, further research is necessary to refine these models and establish their clinical utility. Future work will likely focus on improving model robustness, expanding multi-institutional datasets, and integrating AI tools more seamlessly into clinical workflows. Greater emphasis on prospective studies and demonstration of clinical impact, rather than pure technical performance, will be crucial for meaningful translation into clinical practice.

### 4.1. Downstream Impact on Clinical Care

AI applications have demonstrated potential to positively impact clinical care, ranging from early lesion detection to disease characterization and prognostication. Early and accurate detection of skeletal metastases is critical [83], with important implications in appropriate staging, treatment planning, and prevention of complications such as pathological fractures or spinal cord compression. These systems can potentially improve diagnostic sensitivity and reduce missed metastases, especially in high-volume clinical settings [84].

Another important area of clinical value lies in the ability of AI models to extract high-dimensional imaging features that may reflect underlying tumor biology [85]. Radiomics and deep learning approaches can analyze complex patterns within MRI that are not readily perceptible to human observers [86]. These imaging-derived biomarkers have been explored for tasks such as predicting tumor origin, identifying molecular characteristics (such as EGFR mutation status), and estimating treatment response [87]. If validated in larger and more diverse cohorts, such capabilities may support more personalized oncologic care by providing non-invasive insights into tumor behavior and guiding treatment selection [88].

AI-based predictive models may also contribute to improved risk stratification and clinical decision-making in patients with bone metastases [89]. By integrating imaging features with clinical and laboratory data, predictive algorithms may help identify patients at higher risk of disease progression or adverse treatment complications [90]. Earlier identification of these risks could facilitate more proactive management strategies, including earlier intervention or closer surveillance [7].

### 4.2. Impact on Radiologist Workflow and Decision-Making

From the perspective of the radiologist, increasing demand for advanced imaging has placed substantial pressure on radiology services, particularly in oncologic imaging where MRI examinations are often complex and time-consuming to interpret [91]. The integration of artificial intelligence into the imaging workflow has the potential to alleviate some of these pressures by supporting radiologists in routine and labor-intensive tasks [92]. Automated tools for lesion detection, segmentation, and quantification may assist in rapidly identifying areas of concern, thereby reducing the time required for manual image review and measurement [93]. In the context of bone metastases, where disease may often involve multiple skeletal sites, such tools could be particularly valuable in improving efficiency while maintaining diagnostic accuracy [94].

AI systems may also function as decision-support tools that augment radiologist interpretation. By highlighting suspicious regions or providing probability scores for malignancy, AI models can act as a second reader, helping radiologists identify subtle abnormalities that may otherwise be overlooked [95]. This may be especially useful in complex cases with equivocal findings on MRI [96]. Studies have suggested that AI-assisted interpretation can improve diagnostic performance and help standardize assessments across readers [97], with perhaps the greatest benefit for less experienced readers [98].

Despite these potential benefits, the integration of AI into radiology workflows requires careful consideration. AI tools are most effective when implemented as complementary systems that support, rather than replace radiologist expertise [99]. Although some studies suggest that the efficacy of AI tools in performing cognitive clinical tasks may outperform humans [100], it is important to acknowledge that radiologists remain essential for clinical interpretation and contextual decision-making. As AI technologies continue to mature, their optimal role will likely involve augmenting human expertise, and this can be done through enhancing efficiency whilst improving diagnostic confidence. Ultimately, this allows radiologists to focus on more pressing issues: complex imaging cases and multidisciplinary decision-making [101].

### 4.3. Generalizability and Implementation Challenges

Despite encouraging performance metrics reported in many studies, a key challenge in the adoption of artificial intelligence for bone metastasis assessment on MRI is generalizability [102]. Many AI models are developed and validated using datasets derived from a single institution or a limited number of imaging centers, which may reflect specific scanner types, acquisition parameters, patient populations, or institutional practices [103] and may limit their performance when applied to external populations or different imaging protocols [104]. In addition, the current body of literature remains limited by methodological heterogeneity, a predominance of retrospective study designs, and variability in validation strategies [105]. For the latter, inconsistent use of training, validation, and test datasets across studies complicates interpretation and comparison of reported performance. As a result, models that demonstrate strong performance in controlled research settings may not perform as consistently when applied to external datasets with different imaging protocols or patient demographics [106].

Ensuring robust external validation across diverse clinical environments remains a critical step prior to widespread clinical deployment. This challenge is further compounded by the fact that MRI assessment of bone metastases is highly variable, being influenced by differences in MRI sequences, field strengths, whole-body MRI acquisition protocols, as well as heterogeneity of marrow signal abnormalities [107]. Such variability can substantially affect image appearance and radiomic feature extraction [108]. In clinical practice, MRI protocols often vary across institutions due to differences in equipment, clinical preferences, and resource availability [109]. Addressing this heterogeneity will require the development of models that are robust to these variations, as well as the establishment of standardized imaging and preprocessing pipelines where feasible.

Implementation within real-world clinical workflows also presents practical challenges. Integrating AI tools into existing radiology information systems and picture archiving and communication systems (PACS) requires appropriate technical infrastructure and interoperability [110]. Additionally, regulatory approval, data governance, and patient privacy considerations must be addressed before clinical deployment [111]. These processes are often complex and may delay translation of promising research models into routine clinical use.

Finally, clinician acceptance and trust represent important factors in successful implementation. Radiologists and referring physicians must have confidence in the reliability, interpretability, and clinical relevance of AI outputs. Black-box models that provide predictions without clear explanations may limit clinician adoption [112]. As such, efforts to improve model transparency, provide interpretable outputs, and demonstrate clear clinical benefit will be essential for effective integration of AI models for MRI assessment of bone metastasis into practice.

### 4.4. Limitations of Current Evidence

While the growing body of literature on artificial intelligence applications in MRI for bone metastasis is promising, several limitations within the current evidence base should be acknowledged. A major concern is the predominance of retrospective study designs. Many published studies rely on previously acquired imaging datasets, which may introduce selection bias and limit the ability to fully assess their translational impact [113]. Retrospective analyses often benefit from carefully curated datasets and predefined imaging conditions, which may not accurately reflect the variability and complexity encountered in routine clinical practice.

On the other hand, a limited number of studies have assessed the prospective clinical impact of AI tools on patient outcomes or radiologist performance in real-world settings. Nagendran et al. reported that randomized trial registrations and non-randomized studies comparing the performance of a deep learning algorithm in medical imaging with contemporary expert clinicians found that, of 81 non-randomized clinical trials identified, only nine were prospective and just six were evaluated in real-world clinical settings [114]. While many models demonstrate strong diagnostic or predictive capabilities in experimental environments, the emphasis should be on demonstrating improvements in clinical decision-making, workflow efficiency, or patient outcomes [115]. Addressing these gaps through well-designed prospective and multicenter studies will be important to establish the true clinical value of AI applications in the MRI evaluation of bone metastases.

Finally, the lack of standardized methodological approaches across studies further complicates interpretation of the literature. Manual annotation of skeletal metastases on MRI for training AI models is labor-intensive and time-consuming, often requiring expert radiologist input for lesion delineation. This annotation burden limits the availability of large, high-quality datasets and may contribute to variability in model performance across studies. Differences in imaging sequences, segmentation strategies, feature extraction pipelines, model architectures, and evaluation metrics make direct comparisons between studies challenging [116]. In addition, reporting standards are not always consistent, with some studies providing limited detail on dataset composition, preprocessing, and model validation procedures [117]. These inconsistencies may hinder reproducibility and limit the ability to synthesize findings across the field.

### 4.5. Future Directions and Research Priorities

Looking ahead, several areas of research are needed to further advance the development and clinical integration of artificial intelligence in MRI evaluation of bone metastases. One important priority is the expansion of large well curated datasets [118], enabling studies to capture a wider range of patient populations, scanner types, and imaging protocols. Collaborative data-sharing initiatives may help overcome the limitations of small single-institution datasets and improve the robustness and external validity of AI models. Such efforts would also support more rigorous external validation, which remains a key requirement before clinical implementation.

Another promising direction involves the development of multimodal AI models that integrate imaging data with clinical, laboratory, and genomic information. Bone metastasis is a complex biological process arising from interactions within the bone microenvironment where hematopoiesis, osteogenesis, and osteolysis are tightly regulated [119]. By combining MRI-derived features with additional clinical data, AI systems may provide more comprehensive decision support, including improved lesion characterization, prediction of treatment response, and risk stratification. This approach aligns with broader efforts toward precision oncology, where imaging biomarkers are integrated with other patient-specific data to guide individualized care. While multimodal approaches incorporating clinical and imaging information represent an emerging area of research, their clinical utility in MRI assessment of bone metastases remains to be prospectively established.

In addition, there is growing interest in more generalisable AI frameworks, including multitask and foundation models, which aim to perform multiple imaging tasks within a single architecture [62]. Such approaches may improve scalability and enable more unified analysis across detection, segmentation, and prediction tasks, although their application in MRI remains at an early stage, with further work required to establish their generalisability and clinical utility.

A key priority for advancing MRI-based AI in bone metastases is the establishment of more standardised imaging and validation frameworks. Standardised multi-parameter MRI acquisition protocols should be developed to define core sequences, spatial resolution, slice thickness, and diffusion-weighted imaging parameters, thereby reducing technical heterogeneity between institutions and scanners. Whole body MRI has already benefited from efforts to establish standardised acquisition approaches [120], and these existing frameworks could continue to provide a foundation for developing AI-specific acquisition standards for bone metastasis assessment. In parallel, multicentre collaborative datasets with harmonised imaging protocols, standardised lesion-level annotations, and relevant clinical outcomes should be established, with open-access initiatives where feasible, to facilitate independent benchmarking and external validation of AI models. Taking the Medical Segmentation Decathlon (MSD) as a reference, we have seen how well-curated and standardised imaging datasets can facilitate the development of segmentation algorithms that generalise effectively to previously unseen tasks [121].

A further area that may benefit from future research is the application of artificial intelligence in paediatric oncologic imaging, which remains relatively unexplored [122]. WB-MRI has become an increasingly valuable modality for evaluating metastatic disease in children with malignancies such as neuroblastoma, Ewing sarcoma, and rhabdomyosarcoma. Compared with CT or PET-CT, WB- MRI avoids ionizing radiation exposure, which is particularly important in paediatric populations who may require repeated imaging during staging and treatment monitoring. However, interpretation of marrow changes in children on MRI can be challenging due to age-related physiological changes in marrow composition during skeletal growth. The presence of persistent hematopoietic marrow or marrow reconversion may produce signal patterns that mimic pathological infiltration, making differentiation between normal variation and metastatic disease difficult. AI-based tools that incorporate developmental and anatomical context may therefore have potential to improve lesion detection and reduce diagnostic uncertainty in paediatric MRI.

Future research should also prioritize prospective studies evaluating the impact of AI-assisted interpretation on radiologist performance and clinical workflow. While many algorithms demonstrate strong diagnostic accuracy in retrospective datasets, their real-world value depends on how effectively they can support radiologists in daily practice [123]. Prospective clinical trials and implementation-focused studies should incorporate reader-study designs and predefined clinical-impact endpoints to determine whether AI can ultimately lead to improved patient outcomes.

Finally, greater emphasis should be placed on the interpretability and transparency of AI systems. Radiologists are more likely to trust and adopt AI tools that are not only accurate but also understandable [124]. Continued collaboration between radiologists, data scientists, and clinical researchers will be essential to ensure that AI solutions address meaningful clinical needs and translate into improved care for patients with bone metastases [125]. In practice, future clinical integration may involve AI systems embedded within picture archiving and communication systems (PACS), automatically flagging suspicious skeletal lesions or generating preliminary quantitative tumor burden assessments prior to radiologist review.

## 5. Conclusions

Our review highlights the substantial potential of artificial intelligence (AI) and machine learning to transform MRI evaluation of bone metastases by addressing key challenges across detection, disease characterisation, severity grading, and predictive modelling. We have examined advances across the imaging pathway, including clinical applications, impact on radiologist workflow, and key challenges related to generalisability, implementation, and current evidence limitations. Despite these advances, important gaps remain, particularly in prospective validation, standardisation, and real-world clinical integration. Moving forward, coordinated multidisciplinary collaboration will be essential to harness emerging innovations while addressing these challenges. With continued development and careful implementation, AI has the potential to become an integral component of MRI-based assessment of bone metastases, supporting more accurate, efficient, and personalised patient care.

## Figures and Tables

**Table 1:** Summary of Selected Studies.

No	Study Title	Authorship	Journal Name	Year of Publication	Study Aim	Location of Bone Metastasis	MRI Sequences Used	Artificial Intelligence Technique Used	Key Results
1	Automated Detection and Segmentation of Bone Metastases on Spine MRI Using U-Net A Multicenter	Kim, DH et al. [23]	Korean J Radiol	2024	To develop and evaluate a deep learning model for automated detection and segmentation of bone metastases on spinal MRI.	Spine	T1, CE-T1, CE-T1 (Fat-suppressed)	2D and 3D U-Net	Segmentation Dice 0.699, pixel recall 0.653. Lesion sensitivity 0.828 (internal) and 0.857 (external). Radiologist sensitivity 0.746, PPV 0.701.
2	Machine learning based gray-level co-occurrence matrix early warning system enables accurate detection of colorectal cancer pelvic bone metastases on MRI	Jin, JL et al. [24]	Front Oncol	2023	To develop a Gray-level Co-occurrence Matrix (GLCM)-based radiomics model for predicting bone metastases to support clinical diagnosis and treatment planning.	Pelvic	DWI	neural network model(ANNM), random forest model(RFM), decision tree model(DTM) and support vector machine model(SVMM)	Random forest model AUC 0.926 (training) and 0.919 (validation) for metastasis prediction.
3	A multi-resolution approach for spinal metastasis detection using deep Siamese neural networks	Wang, J et al. [25]	Comput Biol Med	2017	To evaluate the feasibility of automated spinal metastasis detection using deep learning on MRI.	Spine	T2-FS	Deep Siamese Neural Network	Detection True positive rate: 90%; 0.40 false positives per case.
4	Deep learning and transformer-based feature fusion of conventional MRI for differentiating spinal osteolytic bone metastases and multiple myeloma	Bian, HC et al. [26]	Eur J Radiol	2026	To develop a deep learning model integrating conventional MRI and Transformer-based feature fusion to differentiate spinal osteolytic bone metastases from multiple myeloma.	Spine	T1, T2, T2-FS	Transformer-based feature fusion (DenseNet169)	Transformer fusion model AUC 0.783, accuracy 0.723. Best single-sequence (T2-FS) AUC 0.765, accuracy 0.726.
5	Automatic and observable spinal cord compression grading system for lung cancer spinal metastasis	Wang, GY et al. [27]	Quant Imaging Med Surg	2025	To develop an automated system for grading spinal cord compression using multi-tissue segmentation and feature extraction on MRI.	Spine	T2	Unet, ResUnet, ResAgUnet, PSPnet, PSPResUnet, PCAN	Segmentation pixel accuracy 0.819, IoU 0.711. Classification accuracy 0.95, precision 0.89.
6	Improved Prediction of Epidermal Growth Factor Receptor Status by Combined Radiomics of Primary Nonsmall-Cell Lung Cancer and Distant Metastasis	Hu, Y et al. [28]	J Comput Assist Tomogr	2024	To investigate whether MRI-based radiomics features from primary NSCLC and metastatic lesions can predict EGFR mutation status.	Spine	CE-T1	Radiomics	Combined radiomics model AUC 0.936 (training), 0.920 (internal validation), 0.882 (external).
7	Fat Fraction Extracted from Whole-Body Magnetic Resonance (WB-MR) in Bone Metastatic Prostate Cancer: Intra- and Inter-Reader Agreement of Single-Slice and Volumetric Measurements	Agazzi, GM et al. [29]	Tomography	2024	To evaluate the repeatability and reproducibility of fat-fraction measurements on WB-MRI in hormone-naïve prostate cancer patients with bone metastases.	Whole-body	FF%	Radiomics	FF% measurement reproducibility high: intra-reader ICC 0.897–0.971, inter-reader ICC 0.641–0.883. Strong SS–VS correlation (ρ = 0.817).
8	Assessment of Hidden Blood Loss in Spinal Metastasis Surgery: A Comprehensive Approach with MRI-Based Radiomics Models	Zhao, WL et al. [30]	J Magn Reson Imaging	2024	To evaluate MRI-based radiomics models for predicting the risk of intraoperative blood loss in patients undergoing spinal metastasis surgery.	Spine	T1, T2-FS	Radiomics	Fusion radiomics model AUC 0.744. Radscore AUC 0.809.
9	Inter- and Intra-Patient Repeatability of Radiomic Features from Multiparametric Whole-Body MRI in Patients with Metastatic Prostate Cancer	Donners, R et al. [31]	Cancers (Basel)	2024	To assess test–retest repeatability of radiomic features on WB-DWI and T1-weighted Dixon MRI in metastatic castration-resistant prostate cancer bone disease.	Whole-body	T1, DWI, ADC, rFF%	Radiomics	Features that describe extremes in voxel values (minimum, maximum, range, skewness, and kurtosis) showed generally lower ICCs. Several shape-based features demonstrated high inter/intra-patient ICC.
10	Deep learning-based magnetic resonance imaging of the spine in the diagnosis and physiological evaluation of spinal metastases	Wang, DP et al. [32]	J Bone Oncol	2023	To develop a model capable of accurately predicting spinal metastases.	Spine	Not specified	CNN	Prediction model accuracy: 96.45% for spinal metastasis detection.
11	Contrast-enhanced magnetic resonance image segmentation based on improved U-Net and Inception-ResNet in the diagnosis of spinal metastases	Wang, H et al. [33]	J Bone Oncol	2023	To investigate the use of CE-MRI combined with radiomics and deep learning to differentiate spinal metastases from primary malignant spinal tumors.	Spine	T1, T2, T2-FS, CE-T1	U-net, ResNet, Radiomics	U-Net accuracy 0.98 (vs. radiomics 0.74) for differentiating metastasis vs. primary spinal tumors.
12	Identification of Origin for Spinal Metastases from MR Images: Comparison Between Radiomics and Deep Learning Methods	Duan, S et al. [34]	World Neurosurg	2023	To determine whether radiomics and deep learning analysis can identify the primary origin of spinal metastases on MRI.	Spine	CE-T1	Radiomics	Deep Learning model AUC 0.93/0.94 (training), 0.74/0.76 (validation), 0.72/0.76 (external)
13	An MRI-based radiomics nomogram for differentiating spinal metastases from multiple myeloma	Zhang, S et al. [35]	Cancer Imaging	2023	To develop and validate an MRI-based radiomics nomogram for differentiating spinal metastases from multiple myeloma.	Spine	T1, T2-FS	Radiomics	Radiomics nomogram AUC 0.853 (validation), 0.762 (external).
14	Radiomics of Spinal Metastases Originating From Primary Nonsmall Cell Lung Cancer or Breast Cancer and Ability to Predict Epidermal Growth Factor Receptor Mutation/Ki-67 Levels	Niu, SX et al. [36]	J Comput Assist Tomogr	2023	To evaluate spinal MRI radiomics for differentiating metastases from NSCLC and breast cancer and predicting EGFR mutation and Ki-67 expression.	Spine	T1	Radiomics	Training AUC (Ori-RS vs. EGFR-RS vs. Ki-67-RS): 0.890 vs. 0.793 vs. 0.798 Validation AUC (Ori-RS vs. EGFR-RS vs. Ki-67-RS): 0.881 vs. 0.744 vs. 0.738
15	Computer-aided diagnosis of skeletal metastases in multi-parametric whole-body MRI	Ceranka, J et al. [37]	Comput Methods Programs Biomed	2023	To develop a fully automated computer-aided system for detection and segmentation of metastatic bone disease using multiparametric WB-MRI.	Whole-body	3D-T1 FSE or GRE and DWI-MR	U-Net	Detection sensitivity 63%, Dice 0.53
16	Diffusion-weighted MRI radiomics of spine bone tumors: feature stability and machine learning-based classification performance	Gitto, S et al. [38]	Radiol Med	2022	To evaluate the stability of radiomic features and their machine learning classification performance in spinal bone tumors using diffusion- and T2-weighted MRI.	Spine	T2, DWI, ADC	Radiomics	Sensitivity 78%, Specificity 68%, Accuracy 76%, AUC 0.78
17	Vertebral MRI-based radiomics model to differentiate multiple myeloma from metastases: influence of features number on logistic regression model performance	Liu, JF et al. [39]	Eur Radiol	2022	To develop a vertebral MRI radiomics model for differentiating multiple myeloma from spinal metastases, and to evaluate the impact of feature selection on model performance.	Spine	T1, T2	Radiomics	Best model 10EPV (AUC 0.84) outperforms 20EPV (0.71), 15EPV (0.81) and CSF (0.78).
18	Differentiation between spinal multiple myeloma and metastases originated from lung using multi-view attention-guided network	Chen, KL et al. [40]	Front Oncol	2022	To develop a deep learning method for disease differentiation between Multiple Myeloma and metastasis, and to compare its performance with traditional radiomics analysis.	Spine	T1	Multi-view Attention-Guided Network (MAGN)	MAGN model accuracy 0.81, AUC 0.78, F1-score 0.71.
19	Radiomics analysis based on multiple parameters MR imaging in the spine: Predicting treatment response of osteolytic bone metastases to chemotherapy in breast cancer patients	Shi, YJ et al. [41]	Magn Reson Imaging	2022	To evaluate radiomics, apparent diffusion coefficient (ADC), intravoxel incoherent motion (IVIM) and stretched-exponential (SE) MR imaging in prediction of chemotherapy response in patients with spinal metastases.	Spine	DWI, T1, T2, T2-FS	Radiomics	Combined radiomics model AUC 0.905 (training) and 0.873 (validation) for treatment response prediction.
20	Differentiating Between Multiple Myeloma and Metastasis Subtypes of Lumbar Vertebra Lesions Using Machine Learning-Based Radiomics	Xiong, X et al. [42]	Front Oncol	2021	To determine whether machine learning analysis of conventional MRI can differentiate multiple myeloma from metastatic lesions in the lumbar spine.	Spine	T1, T2	Support-Vector Machine (SVM), K-Nearest Neighbor (KNN), Random Forest (RF), Artificial Neural Networks (ANN), and Naïve Bayes (NB) using 10-fold cross-validation	ANN classifier accuracy 0.815, sensitivity 0.879, specificity 0.790 for MM vs. metastasis.
21	Radiomic Machine Learning Classifiers in Spine Bone Tumors: A Multi-Software, Multi-Scanner Study	Chianca, V et al. [43].	Eur J Radiol	2021	To evaluate the use of radiomic features in spinal lesion differentiation.	Spine	T1, T2	Radiomics	ML classification accuracy 94% (internal) and 86% (external) for 2-label classification.
22	Multiparametric MRI-Based Radiomics Approaches for Preoperative Prediction of EGFR Mutation Status in Spinal Bone Metastases in Patients with Lung Adenocarcinoma	Jiang, XR et al. [44]	J Magn Reson Imaging	2021	To develop and validate multiparametric MRI radiomics for preoperative prediction of EGFR mutation in spinal bone metastases.	Spine	T1, T2, T2FS fast SE	Radiomics	EGFR prediction AUC 0.891 (training), 0.771 (validation); sensitivity 0.913, specificity 0.710.
23	Generating Virtual Short Tau Inversion Recovery (STIR) Images from T1- and T2-Weighted Images Using a Conditional Generative Adversarial Network in Spine Imaging	Haubold, J et al. [45]	Diagnostics (Basel)	2021	To develop a conditional GAN model for generating virtual STIR.	Spine	T1, T2, STIR, vSTIR (virtual STIR)	CGAN	vSTIR images equivalent to real STIR in 13/15 categories.
24	Cervical spine osteoradionecrosis or bone metastasis after radiotherapy for nasopharyngeal carcinoma? The MRI-based radiomics for characterization	Zhong, X et al. [46]	BMC Med Imaging	2020	To develop and validate an MRI radiomics nomogram for differentiating cervical spine osteoradionecrosis from metastasis after radiotherapy in nasopharyngeal carcinoma.	Spine	T1	Radiomics	Radiomics nomogram AUC 0.725 (training) and 0.720 (validation).
25	Identification of the most significant magnetic resonance imaging (MRI) radiomic features in oncological patients with vertebral bone marrow metastatic disease: a feasibility study	Filograna, L et al. [47]	Radiol Med	2019	To identify key MRI radiomic features predictive of spinal bone marrow metastases in oncologic patients.	Spine	T1, T2	Radiomics	Best model AUC 0.814 (T1) and 0.912 (T2) for metastasis prediction.
26	Shape, texture and statistical features for classification of benign and malignant vertebral compression fractures in magnetic resonance images	Frighetto-Pereira et al. [48]	Comput Biol Med	2016	To develop a model for classification of vertebral compression fractures on MRI.	Spine	T1	Radiomics	AUC 0.97 for vertebral body fracture detection; AUC 0.92 for discriminating between benign vs. malignant vertebral fractures.
27	Application of Radiomics to the Differential Diagnosis of Temporal Bone Skull Base Lesions: A Pilot Study	Findlay, MC et al. [49]	World Neurosurg	2023	To evaluate radiomic parameters for predicting temporal bone tumor subtype on MRI.	Temporal bone	T1, T2, FLAIR, ADC	Radiomics	A model developed via discriminant analysis using a stepwise algorithm integrating radiomic parameters achieved the best predictive model (*p* = 0.0001).
28	AI-driven software for automated quantification of skeletal metastases and treatment response evaluation using whole-body diffusion-weighted MRI (WB-DWI) in advanced prostate cancer	Candito A et al. [50]	Phys Med Biol	2025	To develop software for automated quantification of total diffusion volume (TDV) and global apparent diffusion coefficient (gADC) on WB-DWI, to assess treatment response in prostate cancer bone metastases.	Whole-body	DWI, ADC	U-Net	Automated lesion delineation Dice 0.60; Total diffusion volume (TDV) ICC ≥ 0.94; response prediction accuracy 80.5%, sensitivity 84.3%, specificity 85.7%.
29	Radiomics and Quantitative MDA Criteria in Breast Cancer with Bone Metastases by MRI: Examples of Calculation Algorithms and Their Practical Use	Steinhauer V et al. [51]	Radiomics Quant MRI Breast Cancer Bone Metast MRI	2023	To improve quantitative and qualitative MRI assessment of metastatic spinal lesions in breast cancer during therapy monitoring.	Spine	T1, T2	Neural Network	Automated analysis detected structural vertebral changes correlating with therapy response.
30	Development and validation of MRI-based radiomics signatures as new markers for preoperative assessment of EGFR mutation and subtypes from bone metastases	Fan Y et al. [52]	BMC Cancer	2022	To develop and externally validate radiomics for identifying EGFR mutations in spinal bone metastases from lung adenocarcinoma on MRI.	Spine	CE-T1	Radiomics	AUCs identifying EGFR mutation 0.851, identifying exon-19 deletion 0.816, identifying exon-21 deletion 0.814 (primary validation). AUCs identifying EGFR mutation 0.807, identifying exon-19 deletion 0.742, identifying exon-21 deletion 0.792 (external validation).
31	Radiomics in Breast Cancer: In-Depth Machine Analysis of MR Images of Metastatic Spine Lesion	Steinhauer V et al. [53]	Sovrem Tekhnol Med	2024	To evaluate software tools for MRI-based advanced analysis of metastatic spinal lesion imaging in breast cancer.	Spine	T1, T2	Radiomics	Pearson correlation coefficient between the classical marker and matrix filter curve = 0.8
32	Deep Learning on MRI Images for Diagnosis of Lung Cancer Spinal Bone Metastasis	Fan X et al. [54]	Contrast Media Mol Imaging	2021	To investigate the application of MRI-based deep learning algorithms for diagnosing spinal bone metastases in lung cancer.	Spine	T2, T2-FS	AdaBoost algorithm and Chan-Vese (CV) algorithm	Dice index = 0.8591 and Jaccard coefficient = 0.8002; superior to OTSU (0.6125 and 0.5541 respectively) and region growing algorithm (0.7293 and 0.6598 respectively).
33	MRI-Based Radiomics Nomogram as a Potential Biomarker to Predict the EGFR Mutations in Exon 19 and 21 Based on Thoracic Spinal Metastases in Lung Adenocarcinoma	Cao R et al. [55]	Acad Radiol	2022	To develop and evaluate an MRI-based radiomics nomogram for differentiating EGFR exon 19 and exon 21 mutations in spinal bone metastases from lung adenocarcinoma.	Spine	T1-FS, T2-FS	Radiomics	Nomogram AUC 0.90 (training), 0.88 (validation)
34	Detection and Segmentation of Pelvic Bones Metastases in MRI Images for Patients With Prostate Cancer Based on Deep Learning	Liu, X et al. [56]	Front Oncol	2021	To develop and evaluate a 3D U-Net model for automated detection and segmentation of pelvic bone metastases in prostate cancer.	Pelvic	DWI, T1	U-Net	Segmentation Dice > 0.85, volumetric similarity (VS) > 0.85, and Hausdorff distance (HD) < 15 mm. Patient-level metastasis detection AUC 0.85 (DWI) and 0.80 (T1). Lesion-level F1-score: 87.6% (DWI) and 87.8% (T1). External M-staging AUC 0.94 (DWI) and 0.89 (T1).

## Data Availability

No new data were created or analyzed in this study. Data sharing is not applicable to this article.

## Supplementary Materials

The following supporting information can be downloaded at: https://www.mdpi.com/article/10.3390/cancers18172794/s1, Table S1: PRISMA checklist.

## References

[1] Macedo F., Ladeira K., Pinho F., Saraiva N., Bonito N., Pinto L., Gonçalves F. (2017). Bone metastases: An overview. Oncol. Rev..

[2] Clézardin P., Coleman R., Puppo M., Ottewell P., Bonnelye E., Paycha F., Confavreux C.B., Holen I. (2021). Bone metastasis: Mechanisms, therapies, and biomarkers. Physiol. Rev..

[3] Onken J.S., Fekonja L.S., Wehowsky R., Hubertus V., Vajkoczy P. (2019). Metastatic dissemination patterns of different primary tumors to the spine and other bones. Clin. Exp. Metastasis.

[4] Tsukamoto S., Kido A., Tanaka Y., Facchini G., Peta G., Rossi G., Mavrogenis A.F. (2021). Current Overview of Treatment for Metastatic Bone Disease. Curr. Oncol..

[5] Cook G.J.R., Thorpe M.P. (2024). Bone Metastases. Cancer J..

[6] O’Sullivan G.J., Carty F.L., Cronin C.G. (2015). Imaging of bone metastasis: An update. World J. Radiol..

[7] Brodowicz T., Hadji P., Niepel D., Diel I. (2017). Early identification and intervention matters: A comprehensive review of current evidence and recommendations for the monitoring of bone health in patients with cancer. Cancer Treat. Rev..

[8] Huang S., Shen Y., Li H., Geng T., Yan J., Wang L., Sui L. (2025). Bone metastases from endocrine cancer: Advances in diagnosis, treatment, and prevention. Front. Endocrinol..

[9] Ouvrard E., Kaseb A., Poterszman N., Porot C., Somme F., Imperiale A. (2024). Nuclear medicine imaging for bone metastases assessment: What else besides bone scintigraphy in the era of personalized medicine?. Front. Med..

[10] Conforti P.M., D’acunto M., Russo P. (2022). Deep Learning for Chondrogenic Tumor Classification through Wavelet Transform of Raman Spectra. Sensors.

[11] Oprea-Lager D.E., Cysouw M.C., Boellaard R., Deroose C.M., de Geus-Oei L.-F., Lopci E., Bidaut L., Herrmann K., Fournier L.S., Bäuerle T. (2021). Bone Metastases Are Measurable: The Role of Whole-Body MRI and Positron Emission Tomography. Front. Oncol..

[12] Lee K.J.X., Tan T.J., Tan E.J., Yan Y.Y. (2023). Magnetic resonance imaging of spinal epidural lesions. Singap. Med. J..

[13] Afnouch M., Bougourzi F., Gaddour O., Dornaika F., Ahmed A.T. (2025). Artificial intelligence in bone metastasis analysis: Current advancements, opportunities and challenges. Comput. Biol. Med..

[14] Caranci F., Tedeschi E., Ugga L., D’Amico A., Schipani S., Bartollino S., Russo C., Splendiani A., Briganti F., Zappia M. (2018). Magnetic Resonance Imaging correlates of benign and malignant alterations of the spinal bone marrow. Acta Biomed..

[15] Wuts J., Ceranka J., Michoux N., Lecouvet F., Vandemeulebroucke J. (2026). Clinically aligned whole-body MRI segmentation of skeletal metastases via Supervised Anatomical Pretraining. J. Bone Oncol..

[16] Szendrői M., Antal I., Szendrői A., Lazáry Á., Varga P.P. (2017). Diagnostic algorithm, prognostic factors and surgical treatment of metastatic cancer diseases of the long bones and spine. EFORT Open Rev..

[17] Woolf D.K., Padhani A.R., Makris A. (2014). Assessing response to treatment of bone metastases from breast cancer: What should be the standard of care?. Ann. Oncol..

[18] Ben Gal O., Soh T.C.F., Vaughan S., Jayasanker V., Mahendra A., Gupta S. (2022). The Prediction of Survival after Surgical Management of Bone Metastases of the Extremities—A Comparison of Prognostic Models. Curr. Oncol..

[19] Bulut H., Demiröz S., Kanay E., Ozkan K., Errani C. (2026). Artificial Intelligence and Machine Learning in Bone Metastasis Management: A Narrative Review. Curr. Oncol..

[20] Gupta A., Parihar P.H., Sheoran M., Gumber S., Singhania S. (2026). Advances in AI and Machine Learning for Diagnostic Imaging of Bone and Soft Tissue Tumors: A Narrative Review. Int. J. Nutr. Pharmacol. Neurol. Dis..

[21] Gupta P., Zhang Z., Song M., Michalowski M., Hu X., Stiglic G., Topaz M. (2025). Rapid review: Growing usage of Multimodal Large Language Models in healthcare. J. Biomed. Inform..

[22] Mehraeen E., Siami H., Montazeryan S., Molavi R., Feyzabadi A., Parvizy I., Masjedlu Z.A., Dehkalani M.N., Mahmoudi S., Ahmadipour A. (2025). Artificial Intelligence Challenges in the Healthcare Industry: A Systematic Review of Recent Evidence. Health Technol. Lett..

[23] Kim D.H., Seo J., Lee J.H., Jeon E.-T., Jeong D., Chae H.D., Lee E., Kang J.H., Choi Y.-H., Kim H.J. (2024). Automated Detection and Segmentation of Bone Metastases on Spine MRI Using U-Net: A Multicenter Study. Korean J. Radiol..

[24] Jin J., Zhou H., Sun S., Tian Z., Ren H., Feng J., Jiang X. (2023). Machine learning based gray-level co-occurrence matrix early warning system enables accurate detection of colorectal cancer pelvic bone metastases on MRI. Front. Oncol..

[25] Wang J., Fang Z., Lang N., Yuan H., Su M.-Y., Baldi P. (2017). A multi-resolution approach for spinal metastasis detection using deep Siamese neural networks. Comput. Biol. Med..

[26] Bian H., Tian N., Zhang Y., Zhou C., Xia X., Miao S., Chai R., Hao D., Cui J. (2025). Deep learning and transformer-based feature fusion of conventional MRI for differentiating spinal osteolytic bone metastases and multiple myeloma. Eur. J. Radiol..

[27] Wang G., Lai G., Wang G., Zhang Y. (2025). Automatic and observable spinal cord compression grading system for lung cancer spinal metastasis. Quant. Imaging Med. Surg..

[28] Hu Y., Geng Y.B., Wang H., Chen H., Wang Z., Fu L.B., Huang B., Jiang W. (2024). Improved Prediction of Epidermal Growth Factor Receptor Status by Combined Radiomics of Primary Nonsmall-Cell Lung Cancer and Distant Metastasis. J. Comput. Assist. Tomogr..

[29] Agazzi G.M., Di Meo N., Rondi P., Saeli C., Volta A.D., Vezzoli M., Berruti A., Borghesi A., Maroldi R., Ravanelli M. (2024). Fat Fraction Extracted from Whole-Body Magnetic Resonance (WB-MR) in Bone Metastatic Prostate Cancer: Intra- and Inter-Reader Agreement of Single-Slice and Volumetric Measurements. Tomography.

[30] Zhao W., Qin S., Wang Q., Chen Y., Liu K., Xin P., Lang N. (2023). Assessment of Hidden Blood Loss in Spinal Metastasis Surgery: A Comprehensive Approach with MRI-Based Radiomics Models. J. Magn. Reson. Imaging.

[31] Donners R., Candito A., Rata M., Sharp A., Messiou C., Koh D.-M., Tunariu N., Blackledge M.D. (2024). Inter- and Intra-Patient Repeatability of Radiomic Features from Multiparametric Whole-Body MRI in Patients with Metastatic Prostate Cancer. Cancers.

[32] Wang D., Sun Y., Tang X., Liu C., Liu R. (2023). Deep learning-based magnetic resonance imaging of the spine in the diagnosis and physiological evaluation of spinal metastases. J. Bone Oncol..

[33] Wang H., Xu S., Fang K.-B., Dai Z.-S., Wei G.-Z., Chen L.-F. (2023). Contrast-enhanced magnetic resonance image segmentation based on improved U-Net and Inception-ResNet in the diagnosis of spinal metastases. J. Bone Oncol..

[34] Duan S., Cao G., Hua Y., Hu J., Zheng Y., Wu F., Xu S., Rong T., Liu B. (2023). Identification of Origin for Spinal Metastases from MR Images: Comparison Between Radiomics and Deep Learning Methods. World Neurosurg..

[35] Zhang S., Liu M., Li S., Cui J., Zhang G., Wang X. (2023). An MRI-based radiomics nomogram for differentiating spinal metastases from multiple myeloma. Cancer Imaging.

[36] Niu S., Zhang H.B., Wang X., Jiang W. (2023). Radiomics of Spinal Metastases Originating From Primary Nonsmall Cell Lung Cancer or Breast Cancer and Ability to Predict Epidermal Growth Factor Receptor Mutation/Ki-67 Levels. J. Comput. Assist. Tomogr..

[37] Ceranka J., Wuts J., Chiabai O., Lecouvet F., Vandemeulebroucke J. (2023). Computer-aided diagnosis of skeletal metastases in multi-parametric whole-body MRI. Comput. Methods Programs Biomed..

[38] Gitto S., Bologna M., Corino V.D.A., Emili I., Albano D., Messina C., Armiraglio E., Parafioriti A., Luzzati A., Mainardi L. (2022). Diffusion-weighted MRI radiomics of spine bone tumors: Feature stability and machine learning-based classification performance. La Radiol. Medica.

[39] Liu J., Guo W., Zeng P., Geng Y., Liu Y., Ouyang H., Lang N., Yuan H. (2022). Vertebral MRI-based radiomics model to differentiate multiple myeloma from metastases: Influence of features number on logistic regression model performance. Eur. Radiol..

[40] Chen K., Cao J., Zhang X., Wang X., Zhao X., Li Q., Chen S., Wang P., Liu T., Du J. (2022). Differentiation between spinal multiple myeloma and metastases originated from lung using multi-view attention-guided network. Front. Oncol..

[41] Shi Y.-J., Zhu H.-T., Li X.-T., Zhang X.-Y., Wei Y.-Y., Yan S., Sun Y.-S. (2022). Radiomics analysis based on multiple parameters MR imaging in the spine: Predicting treatment response of osteolytic bone metastases to chemotherapy in breast cancer patients. Magn. Reson. Imaging.

[42] Xiong X., Wang J., Hu S., Dai Y., Zhang Y., Hu C. (2021). Differentiating Between Multiple Myeloma and Metastasis Subtypes of Lumbar Vertebra Lesions Using Machine Learning–Based Radiomics. Front. Oncol..

[43] Chianca V., Cuocolo R., Gitto S., Albano D., Merli I., Badalyan J., Cortese M.C., Messina C., Luzzati A., Parafioriti A. (2021). Radiomic Machine Learning Classifiers in Spine Bone Tumors: A Multi-Software, Multi-Scanner Study. Eur. J. Radiol..

[44] Jiang X., Ren M., Shuang X., Yang H., Shi D., Lai Q., Dong Y. (2021). MultiparametricMRI-Based Radiomics Approaches for Preoperative Prediction ofEGFRMutation Status in Spinal Bone Metastases in Patients with Lung Adenocarcinoma. J. Magn. Reson. Imaging.

[45] Haubold J., Demircioglu A., Theysohn J.M., Wetter A., Radbruch A., Dörner N., Schlosser T.W., Deuschl C., Li Y., Nassenstein K. (2021). Generating Virtual Short Tau Inversion Recovery (STIR) Images from T1- and T2-Weighted Images Using a Conditional Generative Adversarial Network in Spine Imaging. Diagnostics.

[46] Zhong X., Li L., Jiang H., Yin J., Lu B., Han W., Li J., Zhang J. (2020). Cervical spine osteoradionecrosis or bone metastasis after radiotherapy for nasopharyngeal carcinoma? The MRI-based radiomics for characterization. BMC Med. Imaging.

[47] Filograna L., Lenkowicz J., Cellini F., Dinapoli N., Manfrida S., Magarelli N., Leone A., Colosimo C., Valentini V. (2018). Identification of the most significant magnetic resonance imaging (MRI) radiomic features in oncological patients with vertebral bone marrow metastatic disease: A feasibility study. La Radiol. Medica.

[48] Frighetto-Pereira L., Rangayyan R.M., Metzner G.A., de Azevedo-Marques P.M., Nogueira-Barbosa M.H. (2016). Shape, texture and statistical features for classification of benign and malignant vertebral compression fractures in magnetic resonance images. Comput. Biol. Med..

[49] Findlay M.C., Yost S., Bauer S.Z., Cole K.L., Henson J.C., Lucke-Wold B., Mehkri Y., Abou-Al-Shaar H., Plute T., Friedman L. (2023). Application of Radiomics to the Differential Diagnosis of Temporal Bone Skull Base Lesions: A Pilot Study. World Neurosurg..

[50] Candito A., Blackledge M.D., Holbrey R., Porta N., Ribeiro A., Zugni F., D’eRme L., Castagnoli F., Dragan A., Donners R. (2025). AI-driven software for automated quantification of skeletal metastases and treatment response evaluation using whole-body diffusion-weighted MRI (WB-DWI) in advanced prostate cancer. Phys. Med. Biol..

[51] Steinhauer V., Hartung G. (2024). Radiomics and Quantitative MDA Criteria in Breast Cancer with Bone Metastases by MRI: Examples of Calculation Algorithms and Their Practical Use. Sovrem. Tehnol. Med..

[52] Fan Y., Dong Y., Sun X., Wang H., Zhao P., Wang H., Jiang X. (2022). Development and validation of MRI-based radiomics signatures as new markers for preoperative assessment of EGFR mutation and subtypes from bone metastases. BMC Cancer.

[53] Steinhauer V., Sergeev N. (2022). Radiomics in Breast Cancer: In-Depth Machine Analysis of MR Images of Metastatic Spine Lesion. Sovrem. Tehnol. Med..

[54] Fan X., Zhang X., Zhang Z., Jiang Y. (2021). Deep Learning on MRI Images for Diagnosis of Lung Cancer Spinal Bone Metastasis. Contrast Media Mol. Imaging.

[55] Cao R., Dong Y., Wang X., Ren M., Wang X., Zhao N., Yu T., Zhang L., Luo Y., Cui E.-N. (2022). MRI-Based Radiomics Nomogram as a Potential Biomarker to Predict the EGFR Mutations in Exon 19 and 21 Based on Thoracic Spinal Metastases in Lung Adenocarcinoma. Acad. Radiol..

[56] Liu X., Han C., Cui Y., Xie T., Zhang X., Wang X. (2021). Detection and Segmentation of Pelvic Bones Metastases in MRI Images for Patients with Prostate Cancer Based on Deep Learning. Front. Oncol..

[57] Colombo A., Saia G., Azzena A.A., Rossi A., Zugni F., Pricolo P., Summers P.E., Marvaso G., Grimm R., Bellomi M. (2021). Semi-Automated Segmentation of Bone Metastases from Whole-Body MRI: Reproducibility of Apparent Diffusion Coefficient Measurements. Diagnostics.

[58] Blackledge M.D., Collins D.J., Tunariu N., Orton M.R., Padhani A.R., Leach M.O., Koh D.-M. (2014). Assessment of Treatment Response by Total Tumor Volume and Global Apparent Diffusion Coefficient Using Diffusion-Weighted MRI in Patients with Metastatic Bone Disease: A Feasibility Study. PLoS ONE.

[59] Wang Z., Xiao P., Tan H. (2023). Spinal magnetic resonance image segmentation based on U-net. J. Radiat. Res. Appl. Sci..

[60] Misera L., Müller-Franzes G., Truhn D., Kather J.N. (2024). Weakly Supervised Deep Learning in Radiology. Radiology.

[61] Renard F., Guedria S., De Palma N., Vuillerme N. (2020). Variability and reproducibility in deep learning for medical image segmentation. Sci. Rep..

[62] Tavakoli N., Shakeri Z., Gowda V., Samsel K., Bedayat A., Ghasemiesfe A., Bagci U., Hsiao A., Leiner T., Carr J. (2025). Generative AI and Foundation Models in Radiology: Applications, Opportunities, and Potential Challenges. Radiology.

[63] Hallinan J.T.P.D., Zhu L., Zhang W., Lim D.S.W., Baskar S., Low X.Z., Yeong K.Y., Teo E.C., Kumarakulasinghe N.B., Yap Q.V. (2022). Deep Learning Model for Classifying Metastatic Epidural Spinal Cord Compression on MRI. Front. Oncol..

[64] Spinazzé S., Caraceni A., Schrijvers D. (2005). Epidural spinal cord compression. Crit. Rev. Oncol..

[65] Coleman R.E. (2006). Clinical Features of Metastatic Bone Disease and Risk of Skeletal Morbidity. Clin. Cancer Res..

[66] Varnosfaderani S.M., Forouzanfar M. (2024). The Role of AI in Hospitals and Clinics: Transforming Healthcare in the 21st Century. Bioengineering.

[67] Zhang Y., Li J., Yang Q., Yin S., Hou J., Cao X., Ma S., Wang B., Luo M., Zhou F. (2025). A clinically applicable AI system for detection and diagnosis of bone metastases using CT scans. Nat. Commun..

[68] Sangwon K.L., Han X., Becker A., Zhang Y., Ni R., Zhang J., Alber D.A., Alyakin A., Nakatsuka M., Fabbri N. (2025). Automating the Referral of Bone Metastases Patients With and Without the Use of Large Language Models. Neurosurgery.

[69] Fujibuchi T., Imai H., Kidani T., Miura H. (2022). Effective examination methods for identifying the primary origins of metastatic bone tumors of unknown primary origin during the initial visit: A retrospective chart review study. SAGE Open Med..

[70] Hwang H., Lee S.K., Kim J.-Y. (2021). Comparison of conventional magnetic resonance imaging and diffusion-weighted imaging in the differentiation of bone plasmacytoma from bone metastasis in the extremities. Diagn. Interv. Imaging.

[71] Sanker V., Dawer P., Thaller A., Li Z., Heesen P., Hariharan S., Nordin E.O.R., Cavagnaro M.J., Ratliff J., Desai A. (2025). Artificial Intelligence Models for Predicting Outcomes in Spinal Metastasis: A Systematic Review and Meta-Analysis. J. Clin. Med..

[72] Zhang X., Peng J., Ji G., Li T., Li B., Xiong H. (2023). Research status and progress of radiomics in bone and soft tissue tumors: A review. Medicine.

[73] Qin S., Qu R., Liu K., Yan R., Zhao W., Xu J., Zhang E., Zhou F., Lang N. (2025). Predicting Postoperative Progression of Ossification of the Posterior Longitudinal Ligament in the Cervical Spine Using Interpretable Radiomics Models. Neurospine.

[74] Lecouvet F.E. (2016). Whole-Body MR Imaging: Musculoskeletal Applications. Radiology.

[75] Rockall A.G.F., Li X., Johnson N., Lavdas I., Santhakumaran S., Prevost A.T., Punwani S.F., Goh V.F., Barwick T.D.F., Bharwani N.F. (2023). Development and Evaluation of Machine Learning in Whole-Body Magnetic Resonance Imaging for Detecting Metastases in Patients with Lung or Colon Cancer. Investig. Radiol..

[76] Lecouvet F.E., Chabot C., Taihi L., Kirchgesner T., Triqueneaux P., Malghem J. (2024). Present and future of whole-body MRI in metastatic disease and myeloma: How and why you will do it. Skelet. Radiol..

[77] Rosenkrantz A.B., Cerdas L.C., Hughes D.R., Recht M.P., Nass S.J., Hricak H. (2020). National Trends in Oncologic Diagnostic Imaging. J. Am. Coll. Radiol..

[78] Bendella Z., Widmann C.N., Kindler C., Haase R., Sauer M., Heneka M.T., Radbruch A., Schmeel F.C. (2025). Longitudinal Monitoring of Brain Volume Changes After COVID-19 Infection Using Artificial Intelligence-Based MRI Volumetry. Diagnostics.

[79] Najjar R. (2023). Redefining Radiology: A Review of Artificial Intelligence Integration in Medical Imaging. Diagnostics.

[80] Obuchowicz R., Lasek J., Wodziński M., Piórkowski A., Strzelecki M., Nurzynska K. (2025). Artificial Intelligence-Empowered Radiology—Current Status and Critical Review. Diagnostics.

[81] Korfiatis P., Kline T.L., Meyer H.M., Khalid S., Leiner T., Loufek B.T., Blezek D., Vidal D.E., Hartman R.P., Joppa L.J. (2024). Implementing Artificial Intelligence Algorithms in the Radiology Workflow: Challenges and Considerations. Mayo Clin. Proc. Digit. Health.

[82] Ye Q., Yang H., Lin B., Wang M., Song L., Xie Z., Lu Z., Feng Q., Zhao Y. (2023). Automatic detection, segmentation, and classification of primary bone tumors and bone infections using an ensemble multi-task deep learning framework on multi-parametric MRIs: A multi-center study. Eur. Radiol..

[83] Pu F., Hu Z., Yang Y., Xia P., Xia Z. (2023). Editorial: Diagnosis and treatment of bone metastases. Front. Oncol..

[84] Kukunoor H.R., Andanappa A., Tripathi K.M., Fatima I., Akah O.Z., Faisal A.M., Talat F., Bhatia H., Villalobos A., Dawer P. (2026). Metastatic cancer detection and management with artificial intelligence and augmented reality (Review). Med. Int..

[85] Rajendran A., Rajan R.A., Balasubramaniyam S., Elumalai K. (2025). AI-Enhanced Predictive Imaging in Precision Medicine: Advancing Diagnostic Accuracy and Personalized Treatment. iRADIOLOGY.

[86] McLeod G.A., Stanley E.A.M., Rosenal T., Forkert N.D. (2025). Distinct visual biases affect humans and artificial intelligence in medical imaging diagnoses. npj Digit. Med..

[87] Zafar S., Hafeez A., Shah H., Mutiullah I., Ali A., Khan K., Figueroa-González G., Reyes-Hernández O.D., Quintas-Granados L.I., Peña-Corona S.I. (2025). Emerging biomarkers for early cancer detection and diagnosis: Challenges, innovations, and clinical perspectives. Eur. J. Med. Res..

[88] Ligero M., El Nahhas O.S., Aldea M., Kather J.N. (2025). Artificial intelligence-based biomarkers for treatment decisions in oncology. Trends Cancer.

[89] Li T., Huang H., Zhang S., Zhang Y., Jing H., Sun T., Zhang X., Lu L., Zhang M. (2022). Predictive models based on machine learning for bone metastasis in patients with diagnosed colorectal cancer. Front. Public Health.

[90] Elshimy Y., Alkhatib A.R., Atassi B., Mohammad K.S. (2025). Biomarker-Driven Approaches to Bone Metastases: From Molecular Mechanisms to Clinical Applications. Biomedicines.

[91] Hadi Y.H., Hakami A.M., Ajeebi A.M., Faqihi A.M., Sendi E.T., Masrahi M.A., Alalawi S.A., Musharri F.M., Hakami A.N., Bani-Ahmad M. (2026). Artificial intelligence in radiology workflow: A systematic review into protocol automation and clinical applications. Eur. J. Radiol. Artif. Intell..

[92] Nair A., Ong W., Lee A., Leow N.W., Makmur A., Ting Y.H., Lee Y.J., Ong S.J., Tan J.J.H., Kumar N. (2025). Enhancing Radiologist Productivity with Artificial Intelligence in Magnetic Resonance Imaging (MRI): A Narrative Review. Diagnostics.

[93] Faiella E., Santucci D., Calabrese A., Russo F., Vadalà G., Zobel B.B., Soda P., Iannello G., de Felice C., Denaro V. (2022). Artificial Intelligence in Bone Metastases: An MRI and CT Imaging Review. Int. J. Environ. Res. Public Health.

[94] Sanker V., Gowda P., Thaller A., Li Z., Heesen P., Qiang Z., Hariharan S., Nordin E.O.R., Cavagnaro M.J., Ratliff J. (2025). Applications and Performance of Artificial Intelligence in Spinal Metastasis Imaging: A Systematic Review. J. Clin. Med..

[95] Debellotte O., Dookie R.L., Rinkoo F., Kar A., González J.F.S., Saraf P., Iqbal M.A., Ghazaryan L., Mukunde A.-C., Khalid A. (2025). Artificial Intelligence and Early Detection of Breast, Lung, and Colon Cancer: A Narrative Review. Cureus.

[96] Rud E., Noor D., Galtung K.F., Ottosson F., Jacewicz M., Baco E., Lauritzen P.M. (2022). Validating the screening criteria for bone metastases in treatment-naïve unfavorable intermediate and high-risk prostate cancer—The prevalence and location of bone- and lymph node metastases. Eur. Radiol..

[97] Sabri O., Al-Shargabi B., Abuarqoub A. (2025). The Role of Artificial Intelligence in Improving Diagnostic Accuracy in Medical Imaging: A Review. Comput. Mater. Contin..

[98] Zhou W., Ye Z., Huang G., Zhang X., Xu M., Liu B., Zhuang B., Tang Z., Wang S., Chen D. (2024). Interpretable artificial intelligence-based app assists inexperienced radiologists in diagnosing biliary atresia from sonographic gallbladder images. BMC Med..

[99] Achour N., Zapata T., Saleh Y., Pierscionek B., Azzopardi-Muscat N., Novillo-Ortiz D., Morgan C., Chaouali M. (2025). The role of AI in mitigating the impact of radiologist shortages: A systematised review. Health Technol..

[100] Cabitza F., Campagner A., Ronzio L., Cameli M., Mandoli G.E., Pastore M.C., Sconfienza L.M., Folgado D., Barandas M., Gamboa H. (2023). Rams, hounds and white boxes: Investigating human–AI collaboration protocols in medical diagnosis. Artif. Intell. Med..

[101] Jing A.B., Garg N., Zhang J., Brown J.J. (2025). AI solutions to the radiology workforce shortage. npj Health Syst..

[102] Ehresman J., Lubelski D., Pennington Z., Hung B., Ahmed A.K., Azad T.D., Lehner K., Feghali J., Buser Z., Harrop J. (2021). Utility of prediction model score: A proposed tool to standardize the performance and generalizability of clinical predictive models based on systematic review. J. Neurosurg. Spine.

[103] Xu Y., Li Y., Wang F., Zhang Y., Huang D. (2025). Addressing the current challenges in the clinical application of AI-based Radiomics for cancer imaging. Front. Med..

[104] Kokol P., Kokol M., Zagoranski S. (2022). Machine learning on small size samples: A synthetic knowledge synthesis. Sci. Prog..

[105] Suleman M.U., Mursaleen M., Khalil U., Saboor A., Bilal M., Khan S.A., Subhani M.A., Hussnain M.A., Tabassum S.N., Tahir M. (2025). Assessing the generalizability of artificial intelligence in radiology: A systematic review of performance across different clinical settings. Ann. Med. Surg..

[106] Youssef A., Pencina M., Thakur A., Zhu T., Clifton D., Shah N.H. (2023). External validation of AI models in health should be replaced with recurring local validation. Nat. Med..

[107] Lecouvet F.E., Taihi L., Kirchgesner T., Pasoglou V., Halut M., Sanal H.T., Gitto S., Martín-Noguerol T., Vasilevska-Nikodinovska V., Vanhoenacker F. (2026). ESR Essentials: Bone marrow MRI in oncology—Practice recommendations by the European Society of Musculoskeletal Radiology. Eur. Radiol..

[108] Hagiwara A., Fujita S., Ohno Y., Aoki S. (2020). Variability and Standardization of Quantitative Imaging: Monoparametric to Multiparametric Quantification, Radiomics, and Artificial Intelligence. Investig. Radiol..

[109] Armato S.G., Gruszauskas N.P., MacMahon H., Torno M.D., Li F., Engelmann R.M., Starkey A., Pudela C.L., Marino J.S., Santiago F. (2012). Research Imaging in an Academic Medical Center. Acad. Radiol..

[110] Tejani A.S., Cook T.S., Hussain M., Schmidt T.S., O’donnell K.P. (2024). Integrating and Adopting AI in the Radiology Workflow: A Primer for Standards and Integrating the Healthcare Enterprise (IHE) Profiles. Radiology.

[111] Mennella C., Maniscalco U., De Pietro G., Esposito M. (2024). Ethical and regulatory challenges of AI technologies in healthcare: A narrative review. Heliyon.

[112] Gotta J., Grünewald L.D., Koch V., Mahmoudi S., Bernatz S., Höhne E., Biciusca T., Gökduman A., Wolfram C., Booz C. (2025). Implementation of AI in radiology: The perspective of referring physicians. Insights Imaging.

[113] Koçak B., Ponsiglione A., Stanzione A., Bluethgen C., Santinha J., Ugga L., Huisman M., Klontzas M.E., Cannella R., Cuocolo R. (2024). Bias in artificial intelligence for medical imaging: Fundamentals, detection, avoidance, mitigation, challenges, ethics, and prospects. Diagn. Interv. Radiol..

[114] Nagendran M., Chen Y., Lovejoy C.A., Gordon A.C., Komorowski M., Harvey H., Topol E.J., Ioannidis J.P.A., Collins G.S., Maruthappu M. (2020). Artificial intelligence versus clinicians: Systematic review of design, reporting standards, and claims of deep learning studies. BMJ.

[115] Amusa T., Okunola D., Izinyon O., Alabi A., Akinpeloye O. (2026). Strategies for Embedding Prediction Models in Clinical Decision-Making Workflows. Cureus.

[116] Mashhour M.A., Youssef I., Wahed M.A., Mabrouk M.S. (2025). The Intersection of Genetics and Neuroimaging: A Systematic Review of Imaging Genetics in Neurological Disease for Personalized Treatment. J. Mol. Neurosci..

[117] Kwak S.G., Kim J. (2025). Comprehensive reporting guidelines and checklist for studies developing and utilizing artificial intelligence models. Korean J. Anesthesiol..

[118] Pérez-Sanpablo A.I., Quinzaños-Fresnedo J., Gutiérrez-Martínez J., Rodríguez I.G.L., Roldan-Valadez E. (2025). Transforming Medical Imaging: The Role of Artificial Intelligence Integration in PACS for Enhanced Diagnostic Accuracy and Workflow Efficiency. Curr. Med. Imaging Former. Curr. Med. Imaging Rev..

[119] Esposito M., Guise T., Kang Y. (2017). The Biology of Bone Metastasis. Cold Spring Harb. Perspect. Med..

[120] Parillo M., Mallio C.A. (2025). The Whole-Body MRI Reporting and Data System Guidelines for Prostate Cancer (MET-RADS-P), Multiple Myeloma (MY-RADS), and Cancer Screening (ONCO-RADS). Cancers.

[121] Antonelli M., Reinke A., Bakas S., Farahani K., Kopp-Schneider A., Landman B.A., Litjens G., Menze B., Ronneberger O., Summers R.M. (2022). The Medical Segmentation Decathlon. Nat. Commun..

[122] Davendralingam N., Sebire N.J., Arthurs O.J., Shelmerdine S.C. (2020). Artificial intelligence in paediatric radiology: Future opportunities. Br. J. Radiol..

[123] Lawrence R., Dodsworth E., Massou E., Sherlaw-Johnson C., Ramsay A.I., Walton H., O’REgan T., Gleeson F., Crellin N., Herbert K. (2025). Artificial intelligence for diagnostics in radiology practice: A rapid systematic scoping review. eClinicalMedicine.

[124] Centini F.R., Fedorov D., Arachchige A.S.P.M. (2025). Radiologists’ perspectives on the use of artificial intelligence in emergency radiology: A pilot survey. World J. Radiol..

[125] Bendazzoli S., Persson S., Astaraki M., Pettersson S., Grozman V., Moreno R. (2025). MAIA: A collaborative medical AI platform for integrated healthcare innovation. npj Artif. Intell..

